# Supplementary material for: Why involve families in acute mental healthcare? A collaborative conceptual review
Source: BMJ Open. 2017 Sep 27;7(9):e017680. doi: 10.1136/bmjopen-2017-017680 (PMC5623469; doi:10.1136/bmjopen-2017-017680)
Supplement: Supplementary file 1 [file bmjopen-2017-017680supp001.pdf]

Appendix 1: Search Strategy

Embase/MEDLINE

1. 'caregiver'/exp OR 'caregiver' OR carer*:ab,ti OR (social NEXT/2 network*):ab,ti OR famil*:ab,ti

2. 'psychosis'/exp OR 'psychosis' OR 'bipolar disorder'/exp OR 'bipolar disorder' OR 'mental patient'/exp OR 'mental patient' OR (severe* NEXT/2 mental*):ab,ti AND ill*:ab,ti OR ((serious* NEXT/2 mental*):ab,ti AND ill*:ab,ti) OR (service NEXT/1 user*):ab,ti OR (consumer:ab,ti AND mental:ab,ti) OR 'mental disease'/exp OR 'mental disease'

3. 'mental health care'/exp OR 'mental health care' OR 'crisis intervention'/exp OR 'crisis intervention' OR 'involuntary commitment'/exp OR 'involuntary commitment' OR 'inpatient'/exp OR 'inpatient' OR 'psychiatric department'/exp OR 'psychiatric department' OR acute:ab,ti

4. 'medication therapy management'/exp OR 'medication therapy management' OR 'program development'/exp OR 'program development' OR 'patient care planning'/exp OR 'patient care planning' OR 'health services research'/exp OR 'health services research' OR intervention:ab,ti OR involv*:ab,ti OR program*:ab,ti AND ('psychotherapy'/exp OR 'psychotherapy') OR 'psychotherapy'/exp OR 'psychotherapy'

5. #1 AND #2 AND #3 AND #4

PsycINFO

1. DE “Caregivers” OR MM "Social Networks” OR (DE "Family Members" OR DE "Family”) OR Carer* OR Famil* OR caregiv*

2. DE "Psychosis" OR DE "Acute Psychosis" OR DE "Affective Psychosis" OR DE "Chronic Psychosis" OR DE "Schizophrenia" OR MM "Bipolar Disorder" OR MM "Cyclothymic Personality” OR MM "Mental Disorders” OR ((Severe* OR serious*) AND Mental* AND Ill*) OR (Service AND User*) OR (Consumer* AND Mental)

3. MM "Involuntary Treatment” OR MM "Psychiatric Hospitalization" OR MM "Psychiatric Hospital Admission" OR MM "Psychiatric Hospital Discharge" OR MM "Psychiatric Hospital Readmission" OR DE "Psychiatric Hospital Admission” OR MM "Psychiatric Units” OR MM "Crisis Intervention” OR acute* OR inpatient

4. DE "Treatment Planning" OR DE "Discharge Planning" OR (DE "Intervention" OR DE "Crisis Intervention" OR DE "Early Intervention" OR DE "Family Intervention" OR DE "Group Intervention") OR (MM "Mental Health Services”) OR MM "Program Development” OR involv* OR program* OR interven*

5. #1 AND #2 AND #3 AND #4

AMED

Aysegul Dirik
a.dirik@qmul.ac.uk

exp CAREGIVER/ OR caregiver* OR carer* OR "social network*" OR famil*
AND
PSYCHIATRY AND PSYCHOLOGY/ OR MENTAL DISORDERS/ OR exp PSYCHOTIC DISORDERS/ OR exp SCHIZOPHRENIA/ OR BIPOLAR DISORDER/ OR (severe* AND mental* AND ill*) OR (serious* AND mental* AND ill*) OR "service user*" OR (consumer AND mental)
AND
exp PATIENT CARE PLANNING/ OR exp PATIENT CARE MANAGEMENT/ OR exp METHODS/ OR exp PATIENT ASSESSMENT/ OR exp PATIENT PARTICIPATION/ OR THERAPY/ OR exp PSYCHOTHERAPY/ OR exp FAMILY THERAPY/ OR program* OR intervention* OR invol*
AND
exp MENTAL HEALTH SERVICES/ OR Crisis OR acute* OR exp INPATIENTS/ OR inpatient* OR hospital*

CINAHL

((severe* AND mental* AND ill*) OR (serious* AND mental* AND ill*) OR "service user*" OR (consumer AND mental)).ti,ab
OR
MENTAL DISORDERS/ OR PSYCHOTIC DISORDERS/ OR exp SCHIZOPHRENIA/ OR BIPOLAR DISORDER/
AND
exp *HOSPITALS, PSYCHIATRIC/ OR EXP INPATIENTS/
OR (inpatient* OR Crisis OR acute*).ti,ab
AND
PATIENT CARE PLANNING/ OR PSYCHOTHERAPY/ OR FAMILY THERAPY/
OR (program* OR intervention* OR involv*).ti,ab
AND
(caregiver* OR carer* OR "social network*" OR famil*).ti,ab
OR exp CAREGIVERS/

BNI

exp CAREGIVER/ OR caregiver* OR carer* OR "social network*" OR famil*
AND
PSYCHIATRY AND PSYCHOLOGY/ OR MENTAL DISORDERS/ OR exp PSYCHOTIC DISORDERS/ OR exp SCHIZOPHRENIA/ OR BIPOLAR DISORDER/ OR (severe* AND mental* AND ill*) OR (serious* AND mental* AND ill*) OR "service user*" OR (consumer AND mental)
AND
exp PATIENT CARE PLANNING/ OR exp PATIENT CARE MANAGEMENT/ OR exp METHODS/ OR exp PATIENT ASSESSMENT/ OR exp PATIENT PARTICIPATION/ OR THERAPY/ OR exp PSYCHOTHERAPY/ OR exp FAMILY THERAPY/ OR program* OR intervention* OR invol*
AND
exp MENTAL HEALTH SERVICES/ OR Crisis OR acute* OR exp INPATIENTS/ OR inpatient* OR hospital*

Aysegul Dirik
a.dirik@qmul.ac.uk
